# Characteristics of *Chrysosporium* spp. Pathogens Causing Skin Mycoses in Horses

**DOI:** 10.3390/jof11040297

**Published:** 2025-04-09

**Authors:** Yelena Kukhar, Gulshat Bailina, Ainura Smagulova, Rabiga Uakhit, Vladimir Kiyan

**Affiliations:** 1 Research Platform of Agricultural Biotechnology, S. Seifullin Kazakh Agrotechnical Research University, Astana 010011, Kazakhstan; kucharev@mail.ru (Y.K.); gulshat_010@mail.ru (G.B.); smagulova0114@gmail.com (A.S.); erken.uakhitrabiga@gmail.com (R.U.); 2 Laboratory of Biodiversity and Genetic Resources, National Center for Biotechnology, Astana 010011, Kazakhstan; 3 Scientific Center for Biological Research, Astana 010011, Kazakhstan

**Keywords:** *Chrysosporium*, dermatophytosis, keratinophilic fungi, horses, skin mycoses

## Abstract

Equine skin mycoses are a significant concern in Kazakhstan’s livestock industry due to the country’s historical livestock farming practices, the development of equestrian sports, and food traditions. Skin infections are among the most common fungal infections in horses. Emerging pathogens of equine dermatophytosis include keratinophilic *Chrysosporium* spp., which can degrade and metabolize keratin found in superficial tissues. This, combined with their thermotolerance, contributes to their pathogenicity. In this study, we investigated the biological properties and pathogenicity of two *Chrysosporium* strains isolated from equine skin lesions in northern and central Kazakhstan. Our findings showed that the *Chrysosporium* isolates caused a variety of clinically expressed skin lesions and exhibited cultural and morphological similarities to *Trichophyton mentagrophytes*. Genetic identification using ribosomal gene sequencing revealed 98.9% identity with *Chrysosporium kreiselii* and *Chrysosporium zonatum* sequences in both cases. The *C. kreiselii* strain caused pronounced skin lesions typical of classic dermatomycoses, demonstrated both keratinophilic and keratinolytic properties, and showed resistance to antifungal drugs. In contrast, the *C. zonatum* strain, which caused atypical lesions such as dandruff and seborrhea, was more sensitive to antifungal agents and exhibited keratinophilic properties. Our results highlight the emergence of new pathogenic *Chrysosporium* strains responsible for skin pathology in horses in Kazakhstan. We recommend that the identification of *Chrysosporium* skin infections in horses in Kazakhstan be followed by a comprehensive retrospective analysis of newly identified pathogens, including a full characterization of their pathogenicity.

## 1. Introduction

Fungal infections in horses are generally categorized into superficial mycoses, cutaneous mycoses, subcutaneous infections, and deep (or systemic) mycoses. Cutaneous mycoses are typically localized on the surface of the skin and hooves, such as dermatophytosis and onychomycosis [1,2], and can significantly impact the performance and financial value of the horse [3]. This is especially true for sport horses, as well as horses in Kazakhstan and other countries that are intended for slaughter for food purposes.

Due to the historical characteristics of livestock farming, Kazakhstan’s territory serves as a natural reservoir for classical mycoses in farm animals [4,5,6]. For centuries, breeding and draft horses, cattle, and sheep have been moved from the southern regions to the northern parts of the country or to the highlands (dzhailau) during the summer, and back in the fall. The lack of a system for the treatment and prevention of dermatophytosis has led to the widespread dissemination of pathogens throughout Kazakhstan [4].

The main causative agents of equine dermatophytosis are *Trichophyton equinum*, *Microsporum equinum*, *Trichophyton mentagrophytes*, *Microsporum gypseum*, *Microsporum canis*, and *T. verrucosum* [7,8,9,10,11,12,13]. In addition to dermatomycetes, other fungi responsible for equine diseases include *Aspergillus*, *Alternaria*, and yeast fungi [2,14,15]. Among the most common causative agents of superficial mycoses in horses are yeasts belonging to the genus *Malassezia* [16].

The spectrum of pathogens causing fungal skin infections in horses is expanding and includes mold fungi and yeasts from genera such as *Aspergillus*, *Alternaria*, *Chaetomium*, *Phoma*, *Penicillium*, *Cladosporium*, *Candida*, *Sporothrix*, *Geotrichum*, *Scedosporium*, *Scopulariopsis*, and other representatives [7,13,14,17,18,19,20,21]. This expansion is associated with factors such as global climate change, cosmopolitanism, zoonotic potential, physical inactivity, decreased immune function, and others [2,22,23,24]. The isolation of *Chrysosporium* spp. mold fungi from horses with adiaspiromycosis, corneal lesions, keratitis, and skin mycoses have also been described [25,26,27]. Pathogenic *Chrysosporium*-related fungi (PCRF) have emerged in recent decades as significant pathogens causing mycoses in both captive and free-living reptiles. The recognized pathogenicity of these fungi, along with the high mortality rate in reptiles, makes PCRF a potential threat to other animal groups as well [28].

According to the literature, *Chrysosporium* spp. can cause systemic, subcutaneous, and superficial mycoses in humans and various animals, including horses. The genus *Chrysosporium* belongs to the family Onygenaceae, order Onygenales, class Eurotiomycetes, and phylum Ascomycota. This genus includes about 100 species [29], which are commonly found in the environment, soil, aquatic sediments, as well as on the skin, hair, and nails of animals and humans, and the feathers of birds [30,31,32]. The ability of *Chrysosporium* spp. to degrade and assimilate keratin from superficial tissues classifies them as opportunistic pathogens of cutaneous mycoses and contributes to their pathogenicity [33,34]. Furthermore, the presence of thermotolerance in several keratinophilic *Chrysosporium* spp. further supports their potential pathogenicity [35].

A steady increase in the incidence of *Chrysosporium* spp. infections has been reported in reptiles, causing infectious dermatitis and fatal infections [36,37], as well as in warm-blooded animals, including chickens [38], cats [29], dogs [39,40,41,42], horses [26,27], and other animals. The isolation of *Chrysosporium* spp. from soil, animal hair, or bird feathers has been frequently reported [30,31,43,44,45].

In India, keratinophilic fungi of the genus *Chrysosporium*, including *C. indicum* (26.4%), *C. tropicum* (11.1%), *C. aphanoascus* (2.5%), and *C. arthroderma tuberculatum* (3.4%) [46], have been identified in wild birds and domestic fowls. Additionally, 28 isolates of *Chrysosporium* spp. were found in chickens on the mainland and Nansei Island in Japan [38]. In Germany, a clinical and mycological examination of 500 combs from adult hens yielded 11 isolates of *C. georgiae* [47].

Chrysosporium infections in other animals have been reported much less frequently [29]. Soil-dwelling *Chrysosporium* spp. may be responsible for pulmonary disease in burrowing rodents [48] or cases of adiaspiromycosis with lymph node involvement in wild rabbits (*Oryctolagus cuniculus*) [49]. *C. keratinophilum* was identified in cultures of seven Bennett’s wallabies (*Macropus rufogriseus rufogriseus*) suffering from onychodystrophy, onychomadesis, and severe swelling of the digits on the claws [26].

*Chrysosporium* spp. has been reported isolated from domestic animals [39,40,41,42,43,44]. It was identified as the etiologic agent in one of 10 dogs in Australia [39]. *Chrysosporium* spp. was confirmed as the cause of fungal keratitis in one of 11 dogs [41]. It was also detected in needle aspirates from the iliac lymph nodes and spleen of a German shepherd in Australia [42]. In Turkey, superficial skin lesions caused by *Chrysosporium* spp. were found in two Persian cats and their owner, who lived in the same household [43]. *C. articulatum* was isolated from a cat in Poland with dermatophytosis that resembled that of *Trichophyton* spp. [29]. *Chrysosporium* spp. was also isolated from a horse with a corneal ulcer, stromal abscess, and severe diffuse non-ulcerative keratitis [25]. Two other cases of *Chrysosporium* spp. detection in equine biomaterial were associated with the isolation of the pathogen from horse hair [28,50].

Particular attention should be paid to cases of incorrect diagnosis and pathogen identification in skin mycoses, especially with the emergence of the new pathogen *Chrysosporium* spp., which shares cultural and morphological similarities with classical dermatophytosis pathogens, particularly *T. mentagrophytes* [23,51,52].

The development of a treatment regimen for skin mycoses caused by *Chrysosporium* requires careful attention. The treatment regimens described for warm-blooded animals with superficial mycosis caused by *Chrysosporium* spp. in horses are typically associated with the local use of antifungal drugs, diet, and rest [25,41,42]. In Kazakhstan, treatment of opportunistic skin mycosis is still evolving. No data on the detection of *Chrysosporium* spp. pathogens in horses from Kazakhstan have been found. Generally, information on opportunistic mycoses in Kazakhstan remains fragmentary [53,54,55,56,57], and many opportunistic pathogens are not confirmed by the latest taxonomy [58,59] or genomic analysis [60,61]. This underscores the need for reliable identification of opportunistic pathogens in horses with skin lesions, determining their sensitivity to antimycotics, and developing treatment recommendations for farmers, animal owners, and veterinarians.

The aim of this scientific research was to identify the causative agents of mycosis in horses in northern Kazakhstan and confirm their taxonomic classification. The main objectives of the research are to determine the types of mycosis-causing agents in horses of various purposes in northern Kazakhstan, analyze the occurrence of *Chrysosporium* spp. as a pathogenic agent in the context of global and local research, and describe the taxonomic classification of new causative agents of equine dermatomycosis in Kazakhstan.

## 2. Materials and Methods

### 2.1. Search Strategy

A systematic review and meta-analysis were conducted in accordance with the recommendations of the Preferred Reporting Items for Systematic Reviews and Meta-Analysis (PRISMA). A comprehensive database search was performed using PubMed and the Cochrane Library. The search was limited to English-language articles published between 1964 and 2024.

### 2.2. Research Area and Period

In accordance with the set objectives, we analyzed the pathogens of equine skin mycoses in the northern and central regions of Kazakhstan. Sampling was conducted in three northern regions (Akmola, Kostanay, and North Kazakhstan) and one central region (Karaganda). The study was carried out in the laboratories of the Scientific and Production Platform of Agricultural Biotechnology at Saken Seifullin Kazakh Agrotechnical Research University and the Laboratory of Biodiversity and Genetic Resources of the National Biotechnology Center.

### 2.3. Study Design and Populations

Commercial horses were kept on free grazing, while racehorses and sport horses were housed in individual stalls with controlled grazing. Sampling was conducted in late September to early October. A total of 21 biomaterial samples were collected from animals suspected of being infected or ill, with samples taken from the affected areas on the head and body of the horses. Special attention was given to the affected hair and the presence of skin scales along the edges of the lesions. Scrapings of the dense, white, floury contents from the affected areas were taken with a scalpel at the center of the lesion until the wound surface appeared, following biosafety protocols and humane treatment practices for animals. After collecting samples from the skin and hair, a primary screening was conducted using microscopy to detect the presence of fungal mycelium and spores. Samples were collected using methods approved by the local ethics committee of Seifullin Kazakh Agrotechnical Research University (Protocol No. 2, dated 03 November 2022). The study adhered to the requirements of the International Guiding Principles for Biomedical Research Involving Animals and the European Convention for the Protection of Vertebrate Animals used for Experimental and Other Scientific Purposes [61].

### 2.4. Microbiological Research

Primary isolation of pathogens was performed on Sabouraud media: Sabouraud dextrose agar and Sabouraud medium supplemented with chloramphenicol (Titan Biotech Ltd., Delhi, India) to inhibit the growth of extraneous bacterial biota. Cultivation was carried out at 28 °C for 12–15 days. Pure cultures were isolated on Sabouraud medium without the addition of antimycotics. Inoculation was performed using three passages in a triangular pattern along the diameter of the Petri dishes [62]. Microscopic analysis of the morphological structures of the micromycetes was conducted using a trinocular transmitted light microscope (AxioScope A1, Zeiss, Oberkochen, Germany) at a magnification of ×40 [63].

### 2.5. Biochemical Research

To study the biochemical properties of the isolated fungal cultures, Giss media containing lactose, glucose, sucrose, maltose, and mannitol were used [64]. Christensen agar with 40% urea was employed to test urease activity [65]. Proteolytic activity of the isolated fungi was assessed using skim milk agar. Additionally, nutrient gelatin was used to detect proteolytic activity based on gelatin liquefaction [66]. Columbia nutrient medium with 5% sheep blood agar was used to evaluate the hemolytic activity of the fungi [67]. Keratinolytic properties were assessed using the classic hair perforation test, following standard mycological procedures [55]. Antifungal susceptibility was determined using the CLSI method [68], as described by the authors [69].

### 2.6. Genetic Research

To confirm the accuracy of the cultural and morphological identification, genetic identification of the micromycetes was subsequently performed through ribosomal gene sequencing. Genomic DNA was extracted from five-day-old colonies using the method described by Kukhar et al. (2013) [70]. The procedure for isolating micromycete genomic DNA involved growing the biomass on solid nutrient media for 5–10 days, depending on the colony growth pattern. A colony fragment was separated from the substrate, transferred to a mortar, and 300 μL of 2% CTAB (2% CTAB, 1.4 M NaCl, 20 mM EDTA, 500 mM Tris-HCl, made up to 50 mL with distilled water) was added, followed by incubation with Proteinase K for 14–16 h at 65 °C. DNA extraction continued using the standard phenol-chloroform method [71].

Genotyping was performed using a Bio-Rad T100 thermal cycler (Bio-Rad, Hercules, CA, USA). PCR was carried out in a total reaction volume of 25 μL containing 5 μL of 5× buffer (Promega), 1 μL of dNTPs (20 mM), 1.5 μL of MgCl2 (25 mM), 0.25 U (5 U/μL) of Taq DNA polymerase, 2 μL of primers (20 pmol/μL), and 2 μL of genomic DNA (50 ng/μL). The amplification program included an initial denaturation at 95 °C for 3 min, followed by 35 cycles of denaturation at 95 °C (15 s), annealing at 59 °C (30 s), extension at 72 °C (45 s), and a final extension at 72 °C for 10 min in a SimpliAmp™ Thermal Cycler (Thermo Fisher Scientific, Waltham, MA, USA).

Following amplification, PCR products were electrophoresed on 1.5% agarose gels buffered with 0.5× TBE (4.5 mM Tris, 4.5 mM boric acid, and 1 mM EDTA, pH 8) and stained with ethidium bromide using primers *ITS1* (5′-TCGGTAGGTGAACCTGCGG-3′) and *ITS4* (5′-CCTCCGCTTATTGATATGC-3′) [72]. PCR products were purified enzymatically using ExoI and SAP, and sequences were determined by cycle sequencing. Sequencing was performed with the BigDye^®^ Terminator v3.1 Cycle Sequencing Kit (Applied Biosystems Thermo Fisher Scientific, Carlsbad, CA, USA), following the manufacturer’s instructions, and fragment separation was performed using an IonTorrent automated genetic sequencer (Applied Biosystems Thermo Fisher Scientific, Carlsbad, CA, USA). For comparative analysis, sequences of *Chrysosporium zonatum* strains (OW987038.1, OW986965.1, OW986863.1, KY290545.1) and *Chrysosporium kreiselii* (MN862059.1), published in GenBank (https://blast.ncbi.nlm.nih.gov/Blast.cgi, accessed on 22 December 2024), were used.

### 2.7. Bioinformatics Analysis

For the multiple sequence alignment, the MUSCLE (Multiple Sequence Comparison by Log-Expectation) method was employed. Following the alignment, a phylogenetic analysis was conducted utilizing the maximum likelihood mathematical algorithm implemented in MEGA v. 11 software [73]. The species *Agaricus candussoi* (accession number NR158351) was chosen as the outgroup, providing a clear basis for rooting and enhancing the interpretation of the evolutionary pathways observed in the analysis.

## 3. Results

### 3.1. Data Analysis

An analysis of the literature shows that in recent years, *Chrysosporium* spp. have increasingly been identified as opportunistic pathogens in various invasive systemic and deep non-invasive diseases, as well as in rhinosinusitis and subcutaneous or superficial mycoses in animals and birds (Table 1).

In animals, *Chrysosporium* spp. is most commonly reported to infect cold-blooded animals (various species of snakes, crocodiles, and other reptiles). Cases of *Chrysosporium* spp. being isolated as pathogens have also been recorded in warm-blooded animals. We found only three publications on the detection of *Chrysosporium* spp. from the skin, mucous membranes, or hair of horses.

### 3.2. Clinical Signs and Microbiological Study Results

During our research, in accordance with the set objectives, a total of 90 samples were collected from horses in the northern and central regions of the Republic of Kazakhstan (Table 2).

As shown in Table 2, the total number of positive samples was 68.9% (62 samples). In 31.1% of cases, no pathogen growth was detected in the total number of samples. As a result of the cultural mycological analysis of the biological material, fungi were isolated from 62 samples. Of the total isolates, 14.4% were classical dermatophytes, while 85.6% were opportunistic fungi, including 77.8% mold fungi and 7.8% yeast strains.

**Figure 1:**
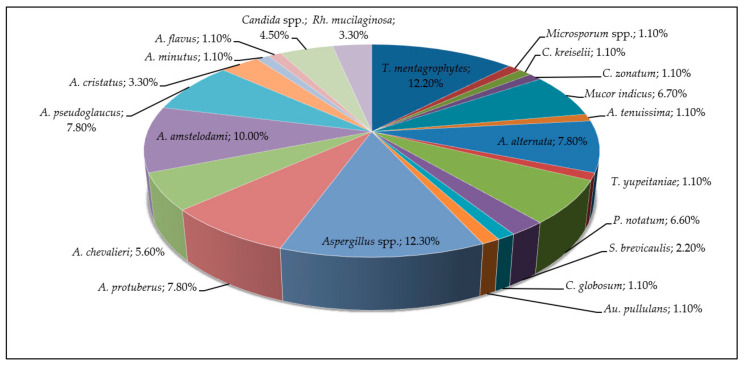
Taxonomic composition and percentage of fungi isolated from affected areas of horse skin in the northern and central regions of Kazakhstan (2023–2024).

A total of 90 isolates were obtained from the positive samples, and 26 fungal taxa were identified at the genus level (Figure 1).

In the mycobiota of skin lesions of horses with various purposes, filamentous fungi of the genus *Aspergillus* dominated, comprising 49% of the total. The breakdown of *Aspergillus* species was as follows: *Aspergillus* spp.—12.3%, *A. protuberus*—7.8%, *A. chevalieri*—5.6%, *A. amstelodami*—10.0%, *A. pseudoglaucus*—7.8%, *A. cristatus*—3.3%, *A. minutus*—1.1%, and *A. flavus*—1.1%. Other common species included *A. alternata*—7.8%, *Penicillium notatum*—6.6%, and *Mucor indicus*—6.7%. Additionally, several yeast species were identified: *Rhodotorula mucilaginosa*—3.3%, yeasts of the genus *Candida*—4.5%, and *Aureobasidium pullulans*—1.1%. Among keratinophilic fungi, *T. mentagrophytes* was isolated—12.2%, *Microsporum* spp.—1.1%, and *Chrysosporium* spp.—2.2%. In total, keratinophilic fungi accounted for 15.5%.

**Figure 2:**
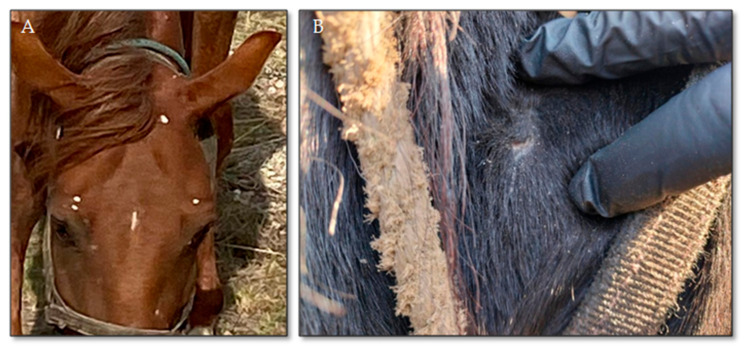
Appearance of skin lesions in horses with mycosis caused by *Chrysosporium* spp.: (**A**) lesions on the horse’s head, (**B**) scaling lesions.

During our scientific research on the identification of dermatophytosis pathogens in horses in the Northern and Central regions of Kazakhstan, we found that some horses exhibited various skin lesions. Some of these lesions showed clinical similarities to those characteristics of classic dermatomycosis, while others presented uncharacteristic signs (Figure 2). The lesions were located on the muzzle, neck, and body of the animals, with the majority of the detected lesions on the muzzle. The lesions were primarily small, white, and mealy, measuring 1–2 cm in diameter. The alopecia zones on the facial areas of the horses’ heads were characterized by lesions measuring 2.0 × 0.9 cm, with clear, even, oval-shaped borders. The skin within the affected area appeared hyperemic (Figure 2).

As shown in Figure 2, the lesions on the commercial horse (Figure 2A) resembled classic dry trichophytosis, characterized by a white powdery surface. The flaking areas on the racehorse’s body (Figure 2B) were clinically identified as dry dermatitis with dandruff formation, though seborrhea remains questionable.

Primary isolation and characterization of the pathogens revealed the presence of various types of colonies. During the initial isolation from samples, fluffy brown colonies with black pigment formation were observed, along with whitish to light beige colonies that formed pigmentation foci ranging from yellowish to orange and resembled dermatophyte colonies morphologically.

**Figure 3:**
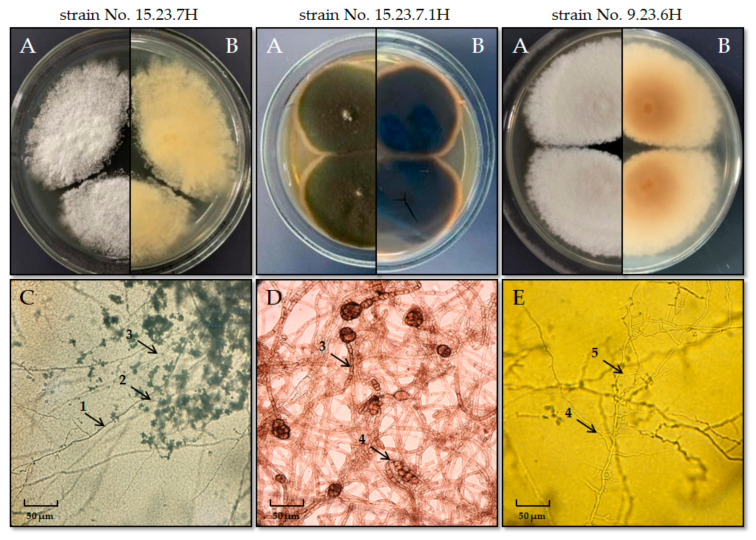
External appearance of colonies from pure cultures isolated from horse biomaterial on the 15th day of cultivation: (**A**)—front view and (**B**)—rear view of the colony; (**С**)—microscopic analysis of strain 15.23.7H; (**D**)—features of the microscopic structure of strain 15.23.7.1H; (**E**)—features of the mycelium structure of strain 9.23.6H; smooth mycelium (1), cluster-shaped microconidia (2), septate mycelium (3), macroconidia (4), microconidia (5).

When analyzing pure cultures, attention was given to the color of both the front and back sides of the colonies, substrate pigmentation, and the shape and surface of the colonies (Figure 3).

Colonies of pure cultures from strains No. 15.23.7H and No. 9.23.6H, which resemble dermatophytes, are uniform and exhibit distinct zonality: a powdery raised center, a fluffy growing edge, and a light yellow or pale orange pigment on the reverse side of the colony, although they differ in size (Figure 3). Cultural and morphological identification suggested that the white colonies with straw-yellow and orange-brown pigments correspond to dermatophyte colonies of the genus *Trichophyton*. The morphological similarity of the strain colony to typical colonies of *T. mentagrophytes*, along with the presence of microstructures such as twisted, colorless, thin, smooth or branched septate mycelium with chlamydospores and microconidia in characteristic single or cluster-like formations (Figure 3C,E), allowed for the identification of this pathogen as *T. mentagrophytes*.

In contrast, the pure culture of primary heterogeneous brown colonies from sample No. 15.23.7.1H differed significantly (Figure 3). These colonies had a brown color on the front, a black pigment on the reverse side, did not stain the substrate, and were identified by cultural and morphological characteristics as fungi of the genus *Alternaria*. Microscopic analysis of strain No. 15.23.7.1H revealed the presence of dark brown mycelium and conidia characteristic of *Alternaria alternata* (Figure 3D).

**Figure 4:**
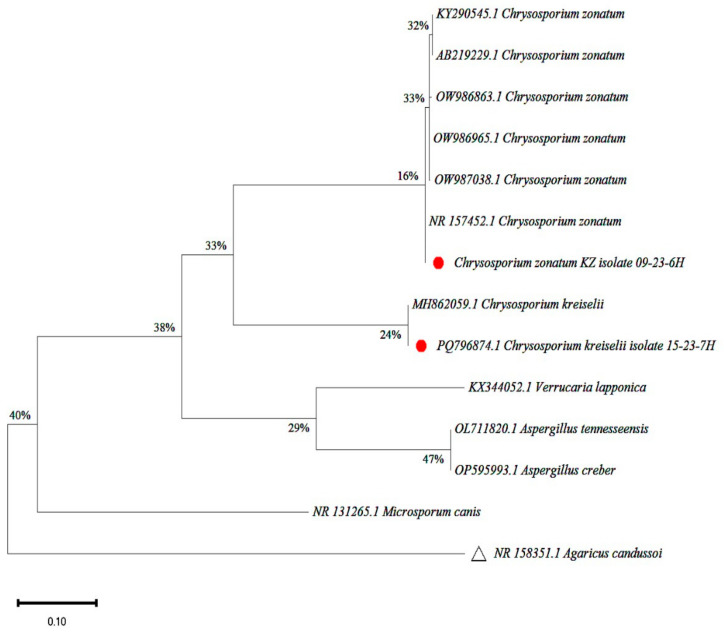
Maximum likelihood phylogenetic tree (divergence = 0.10).

The obtained sequence was analyzed using BLAST (Stable release 2.16.0+[1], 25 June 2024) with the NCBI database, which revealed 98.9% identity in both cases with the sequences of *C. kreiselii* and *C. zonatum*. Phylogenetic analysis was performed on the obtained data for the *Chrysosporium* strains. The final dataset contained 1898 positions. Two isolates from this study (marked with red circles), designated as *C. zonatum* isolate KZ 09-23-6H and *C. kreiselii* isolate 15-23-7H, grouped with their respective reference species (*C. zonatum* and *C. kreiselii*), confirming their taxonomic placement. *Agaricus candussol* (NR 158351.1) served as an outgroup species, providing evolutionary context. Other fungal species also formed a separate clade with their related sequences. Low node support is evident, indicating a moderate but weak level of clustering. The phylogenetic tree of species evolution, constructed using the maximum likelihood algorithm, is presented in Figure 4.

After analyzing the obtained results, the nucleotide sequence of *C. kreiselii* was deposited in the GenBank database under accession number PQ796874, and that of *C. zonatum* was deposited under accession number PV034795.

Thus, isolates from horses with dermatological lesions, initially diagnosed as *Trichophyton* spp. infection, were identified by sequencing as *Chrysosporium* spp., which are generally considered non-pathogenic fungi.

### 3.3. Study on the Pathogenicity of Isolated Strains

**Figure 5:**
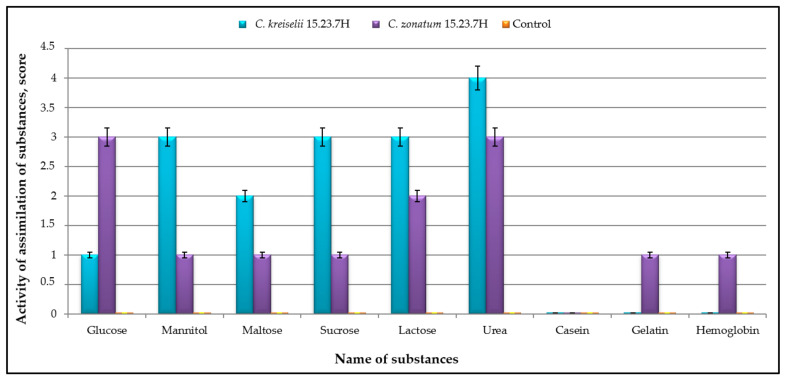
Results of the biochemical test for the enzymatic activity of *C. kreiselii* No. 15.23.7H and *C. zonatum* No. 9.23.6H, isolated from horse biomaterial.

To confirm the pathogenicity of potential opportunistic strains, enzymatic activity, including activity against keratin, was assessed. Analysis of the enzymatic activity of *C. kreiselii* strain No. 15.23.7H and *C. zonatum* strain No. 9.23.6H, isolated from horse biomaterial samples, revealed that both isolates were capable of breaking down glucose, mannitol, maltose, sucrose, lactose, and urea. *C. kreiselii* No. 15.23.7H showed higher saccharolytic activity, though it was inferior to *C. zonatum* No. 9.23.6H only in glucose degradation. Proteolytic enzyme activity toward gelatin and hemoglobin was observed in *C. zonatum* No. 9.23.6H. Neither strain broke down milk casein (Figure 5).

**Figure 6:**
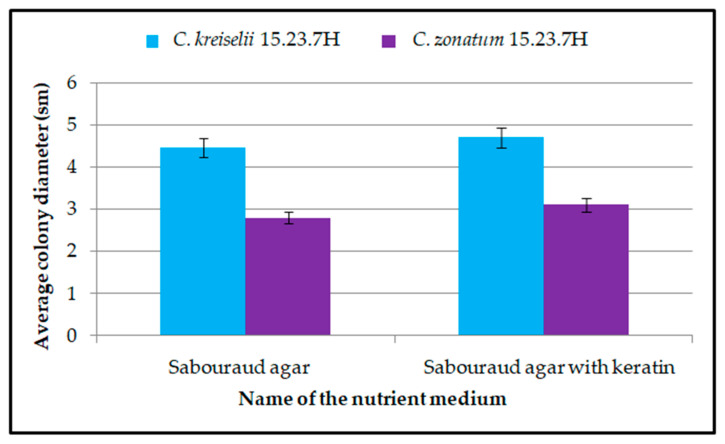
Keratinophilic properties of *C. kreiselii* and *C. zonatum* strains.

When determining the nature of growth on media enriched with keratin, both isolates demonstrated the ability of the micromycetes to assimilate this protein, as evidenced by an increase in colony diameter and visible growth of both surface and deep mycelium (Figure 6).

As shown in Figure 6, colonies of *Chrysosporium* spp. strains on nutrient dextrose Sabouraud agar enriched with keratin had a larger diameter compared to colonies growing on regular Sabouraud agar, which served as the control. This result indicates the presence of keratinophilic properties in *C. kreiselii* No. 15.23.7H and *C. zonatum* No. 9.23.6H.

**Figure 7:**
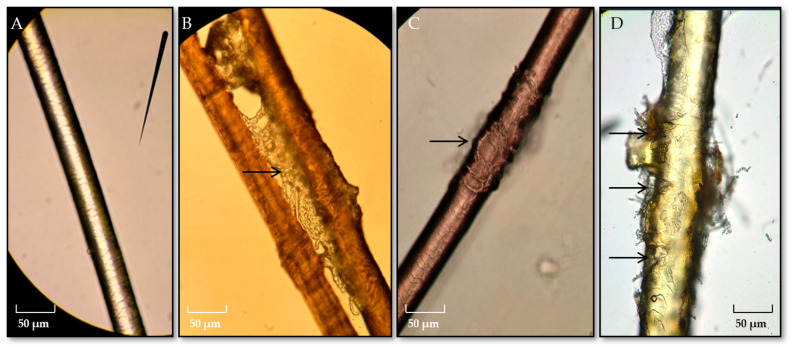
Hair deformation under the influence of *Chrysosporium* spp. strains: (**A**)—control, (**B**)—mycelial growth on the hair surface, (**C**)—hair cuticle damage, (**D**)—hair keratinolysis.

In the hair perforation test, the analyzed *Chrysosporium* spp. strains exhibited keratinolytic properties. More pronounced keratinolytic activity was observed in *C. kreiselii* №15.23.7H, which manifested as abundant mycelial growth on the hair surface (Figure 7A), damage to the hair cuticle externally visible as surface erosion (Figure 7B), loosening or thinning of the hair, and noticeable keratinolysis, with visible damage resembling “pegs” (Figure 7C).

### 3.4. Antifungal Drug Susceptibility

**Figure 8:**
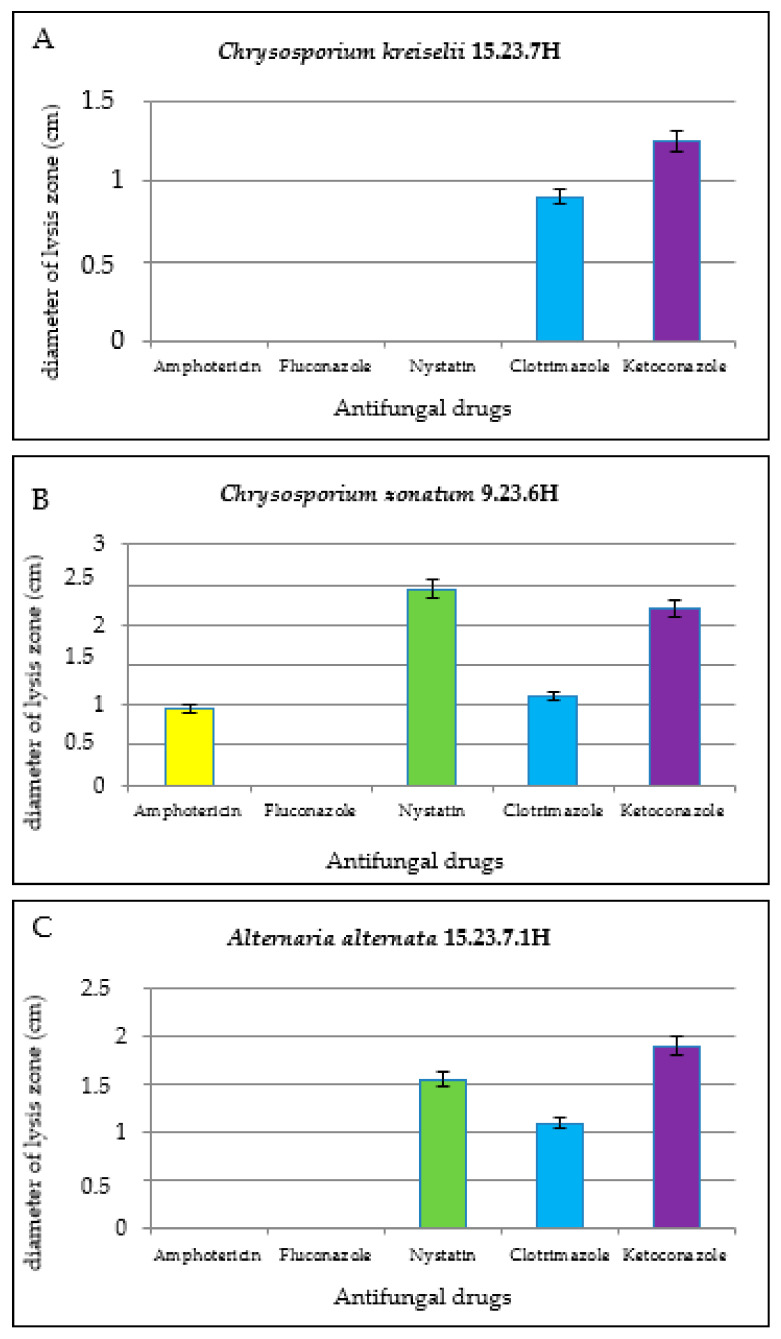
Comparative sensitivity of fungal strains isolated from horses to antifungal drugs: (**A**)—*C. kreiselii* strain No. 15.23.7H; (**B**)—*C. zonatum* strain No. 9.23.6H; (**C**)—*A. alternata* strain No. 15.23.7H.

Analysis of the susceptibility of fungal strains isolated from horses to antifungal drugs showed that the *C. kreiselii* strain 15.23.7H (Figure 8A), isolated from a racehorse in the Karaganda region, is resistant to almost all antifungal drugs, while the *C. zonatum* strain 9.23.6H is susceptible to all drugs except Fluconazole (Figure 8B). The *A. alternata* strain No. 15.23.7.1H (Figure 8C), isolated in association with *C. kreiselii* 15.23.7H, is susceptible to Ketoconazole, weakly sensitive to Nystatin, and Clotrimazole (Figure 8).

The *C. kreiselii* 15.23.7H strain is resistant to Nystatin, Amphotericin, and Fluconazole, and shows weak sensitivity to Clotrimazole and Ketoconazole. The *C. zonatum* 9.23.6H strain, isolated from a racehorse in the Karaganda region, showed weak sensitivity to Amphotericin, was sensitive to Nystatin and Ketoconazole, and resistant to Clotrimazole and Fluconazole.

## 4. Discussion

During the initial isolation of pathogens from 543 samples of biological material from domestic and wild animals in Northern Kazakhstan, conducted between 2012 and 2022, the predominant micromycetes were opportunistic mold fungi (50.2%), including *Mucor* spp., *Penicillium* spp., *Aspergillus* spp., *Alternaria* spp., *Chaetomium* spp., *Eurotium* spp., *Phoma* spp., *Trichoderma* spp., *Lecanicillium psalliotae*, *Scopulariopsis brevicaulis*, and others. Dermatophytes of the genera *Trichophyton* spp. and *Microsporum* spp. were detected in only 17.1% of cases. Yeasts of the genera *Candida* spp., *Rhodotorula* spp., and *Exophiala* spp. were isolated in only 5.1% of cases. In 27.6% of cases, no growth of micromycetes was observed in the biomaterial samples, or bacterial growth was noted. In the regions of Northern Kazakhstan and Western Siberia in Russia, a shift toward an increase in the number of opportunistic microorganisms was observed. Of the total number of identified micromycetes, 23.6% were classic causative agents of dermatomycosis, 69.3% were opportunistic causative agents of mold mycoses, and 7% were causative agents of yeast mycoses [97,98]. Our data are consistent with those of other researchers who have reported an increase in the proportion of opportunistic pathogens worldwide [2,7,13,14,15,16,17,18,19,20,21,99,100,101,102].

An analysis of the literature on cases of cutaneous mycoses caused by *Chrysosporium* spp. revealed reports of cutaneous infections in humans [99,100,101,102], reptiles, both wild and captive [74,75,76,77,78,79,80,81,82,83,84,85,86,87,88,89,90,91,92,93,94,95,103,104], chickens [37], dogs [40], kangaroos [43], and cats [30,97]. Publications also describe the isolation of *Chrysosporium* spp. from horse hair [28,51]. To date, there have been no reports on the detection of *Chrysosporium* spp. in biomaterial or cases of cutaneous mycoses caused by *Chrysosporium* spp. in farm, wild, or domestic animals in Kazakhstan. However, when isolating pathogens from wild, farm, and domestic animals in Kazakhstan between 2023 and 2025, the authors of this article identified the emergence of new opportunistic microorganisms—representatives of the mold mycobiota, including *Chrysosporium* spp., *Corynascus novoguineensis*, and *Lachnum controversum* [105].

A racehorse from central Kazakhstan was found to have skin lesions similar to those characteristics of classic dermatomycosis. A farm horse from northern Kazakhstan showed uncharacteristic lesions, such as dandruff and seborrheic lesions.

Primary fungal cultures of pathogens isolated from the affected horses resembled dermatophytes and were thus cultured as pure strains. Colonies of two strains of *Chrysosporium* spp., isolated from the biomaterial of racehorses and farm horses, were initially identified based on their primary cultural and morphological features as dermatophytes of the genus *Trichophyton*. Moreover, the cultural, morphological, and biochemical characteristics of these colonies were consistent with *T. mentagrophytes*. The search for microconidia and macroconidia was challenging, as their formation was not observed in young colonies.

A similar case of laboratory misidentification based on culture-morphological identification was described by Kizerwetter-Świda et al. (2024) [29], in which *Chrysosporium* spp. were isolated from a cat with dermatological lesions typical of dermatophytosis caused by *Trichophyton* spp. The authors noted that the dermatological lesions in the cat were clinically consistent with dermatophytosis usually caused by *Trichophyton* spp., including alopecia on the back of the neck, ventral abdomen, and hind limbs. Initial identification of the pathogen, based on phenotypic properties, indicated *Trichophyton* spp., but this was excluded by MALDI-ToF MS. Ultimately, the correct identification of the strain as *C. articulatum* was confirmed through ribosomal gene sequencing [9].

In our study of the keratinophilic properties of pathogens during cultivation on a modified Sabouraud medium enriched with animal keratin, we found that both strains of *Chrysosporium* spp., isolated from the biomaterial of a racehorse and a farm horse from central and northern Kazakhstan, exhibited keratinophilic properties. This was evidenced by the more rapid colony formation and biomass accumulation on Sabouraud media containing keratin. Previous studies have reported the isolation of keratinophilic fungi of the genus *Chrysosporium* from soil, animal hair, and bird feathers [42,44,45]. For instance, fifteen keratinophilic fungi, including eight species of *Chrysosporium*, predominantly *C. zonatum*, were isolated from the hair of cows and buffaloes, soil from fields, and animal cages [44]. In India, representatives of the genus *Chrysosporium* were isolated from feather samples of 117 birds representing 11 species examined for keratinophilic fungi: *C. indicum* (26.4%), *C. tropicum* (11.1%), *C. aphanoascus* spp. (2.5%), and *C. arthroderma tuberculatum* (3.4%) [45].

As is well known, the pathogenic properties of skin mycosis pathogens are primarily attributed to the activity of the keratinase enzyme and their ability to degrade keratin [106]. In pure cultures, a positive hair perforation test was observed, demonstrating pronounced keratinolytic properties. This included abundant mycelium growth on the hair surface, damage to the hair cuticle, corrosion of the hair surface, loosening and thinning of the hair, and the appearance of noticeable “pegs”, although not characteristic of *T. mentagrophytes*.

It is important to note that strain *A. alternata* No. 15.23.7.1H, isolated from horse biomaterial in association with *C. kreiselii* 15.23.7H, did not display pronounced keratinolytic properties. *A. alternata* No. 15.23.7.1H is a keratinophilic fungus, as it grew abundantly on the hair surface in vitro, without forming characteristic “pegs” and causing minimal damage to the cuticle, manifesting as longitudinal cracks on its surface. Based on this observation, the diagnosis was initially dermatophytosis caused by *T. mentagrophytes*. However, molecular genetics identification of the strains revealed 98.9% identity with the *C. kreiselii* sequence and 98.9% identity with the *C. zonatum* sequence, allowing us to exclude the diagnosis of *T. mentagrophytes*.

Furthermore, it is important to note that several geophilic *Chrysosporium* spp., including *Anixiopsis stercoraria*, *C. keratinophilum*, *C. tropicum*, *C. pannorum*, *C. curreyi*, *A. multifidum*, and *A. tuberculatum*, were reported by Chabasse D. et al. (1989) [107] to not exhibit actual hair contamination during pathogenicity assessments. While the authors did not confirm the pathogenic role of these keratinophilic fungi, they noted that their ability to remain viable for several weeks in skin and abdominal tissue suggested that they could become pathogenic under certain conditions [46].

The determination of the susceptibility of *Chrysosporium* strains isolated from horses to antifungal drugs revealed that both strains are resistant to fluconazole. Specifically, the *C. kreiselii* strain 15.23.7H, isolated from a racehorse in the Karaganda region, is resistant to nearly all the studied antifungals, while the *C. zonatum* strain 9.23.6H is susceptible to all drugs except fluconazole. Our findings are consistent with those of authors who have discussed the global issue of dermatophyte and mold fungi resistance to antifungal drugs [108].

It is also important to note that no information exists regarding the successful treatment of cutaneous forms of equine mycoses caused by *Chrysosporium* spp. using antifungal drugs. We hypothesize that this may be linked to the morphological similarity of *Chrysosporium* spp. to dermatophytes of the *Trichophyton* genus, especially *T. mentagrophytes*, as reported earlier [1,10,24]. This similarity may have led to misdiagnosis and inappropriate treatment strategies.

We argue that the presence of keratinophilic and keratinolytic properties, combined with resistance to antimycotics, are significant factors supporting the pathogenicity of *Chrysosporium* strains. This suggests that these strains did not merely colonize the horse’s coat accidentally but actually caused skin pathology in the horses. A similar case of *Chrysosporium* spp. isolation from horses was described by Khalaf et al. (2024) [50]. Our data on the identification of *Chrysosporium* strains align with the viewpoint of specialists [103], who believe that the low frequency of reported primary skin infections caused by *Chrysosporium* may reflect either an underestimation of this diagnosis in the literature or a misidentification of this fungus as a more commonly encountered species with similar morphology.

We conclude that detecting *Chrysosporium* skin infections in horses in Kazakhstan requires a thorough retrospective analysis of the spectrum of newly identified pathogens, with a comprehensive characterization of the pathogenicity of the strains. Accurate identification of the pathogen, along with an assessment of pathogenicity factors and sensitivity to antimycotics, will help develop effective treatment and prevention strategies, minimizing the risk of medical errors in the management of equine skin mycoses associated with *Chrysosporium* spp.

## 5. Conclusions

*Chrysosporium* spp. were isolated from lesions on the skin of horses in Kazakhstan for the first time. Molecular genetics identification of the strain revealed a 98.9% identity with the sequences of *C. kreiselii* and *C. zonatum* in each case. The *C. kreiselii* strain was isolated from horse biomaterial in association with the opportunistic pathogen strain *A. alternata* No. 15.23.7.1H, while the *C. zonatum* strain was isolated in monoculture. The *C. kreiselii* and *C. zonatum* strains were distinguished by their pronounced pathogenicity, keratinophilic properties, and resistance to antifungal drugs. Both strains are resistant to Fluconazole. The *C. kreiselii* 15.23.7H strain is also resistant to Nystatin and Amphotericin, and is weakly susceptible to Clotrimazole and Ketoconazole. The *C. zonatum* 9.23.6.H strain is weakly susceptible to Amphotericin and is resistant to Clotrimazole and Fluconazole.

## Figures and Tables

**Table 1:** Pathogens of *Chrysosporium* spp. and the pathologies they cause.

Isolated Fungus	Object	Source	Immunosuppression	Treatment	Reference
*Chrysosporium* infection of reptiles					
*Chrysosporium* spp.	Reptiles, green iguanas (*Iguana iguana*), eastern horned rattlesnakes (*Sistrurus catenatus*)	fungal brain abscess, necrotizing dermatomycosis, deep ulcers, necrosis	conditions of detention	itraconazole	[74,75]
*Chrysosporium* spp., *Nannizziopsis vriesii*	bearded dragons (*Pogona vitticeps*), green iguanas (*Iguana iguana*), snakes (various species), inland dragon (*Pogona vitticeps*), tentacled snakes (*Erpeton tentaculatum*), tree snakes (*Boiga irregularis*), saltwater crocodiles (*Crocodylus porosus*)	vesicular lesions and bullae, necrosis, scaling, and ulceration, progressing to involve muscles and bones deep mycosis, necrotizing fungal dermatitis, cutaneous hyalohyphomycosis	poor living conditions and stress	itraconazole, ketoconazole, voridazole	[76,77,78,79,80,81,82,83,84,85,86,87,88,89]
*N. vriesii*, *C. guarroi*, *C. queenslandicum*, *C. ophiodiicola*	chameleons, reptiles, tentacle snakes (*Erpeton tentaculatum*), dragon (*Pogona vitticeps*), bearded dragon (*Pogona vitticeps*), *Thamnophis* snakes, green iguanas (*Iguana iguana*)	systemic lesions, necrosis and soft tissue lesions, ulcers and infiltration in the skin, widespread infection, including both skin lesions and lesions of internal organs	general weakness and anorexia, progressive emaciation, necrosis, and death of the animal, the infection may spread to deep tissues and become systemic	ketoconazole, itraconazole, voriconazole, itraconazole	[82,90,91,92,93,94,95]
*Chrysosporium* infection of warm-blooded animals and birds					
*C. tropicum*	chickens	dermatomycosis, mycosis of the comb of chickens	physical inactivity, crowded conditions	not indicated	[37]
*Chrysosporium* spp.	dog	fungal infection, signs of multiple foci of discospondylitis	breed predisposition	itraconazole	[39]
*C. evolceanui*	9-year-old miniature pinscher dog	chrysosporiasis of the skin	localized exudative epidermitis on the neck and both sides of the croup, skin hyperpigmentation, hormonal disorders	cetavlon	[39]
*Chrysosporium* spp.	dogs—11 pieces	fungal keratitis	genetic predisposition	antifungal drugs, euthanasia	[41]
*Chrysosporium* spp.	German shepherd	Disseminated *Chrysosporium* infection	lesions of the iliac lymph nodes and spleen	posaconazole	[43,96]
*C. articulatum*, *Chrysosporium* spp.	cat cats—2 pieces	trichophytosis, dermatophytosis, superficial skin lesions	food allergy, host-borne infection	diet	[29,43]
*C. keratinophilum*	kangaroo, Bennett’s wallaby	onychomycosis of the claws	onychodystrophy, onychomadesis, severe digital tumor	ketoconazole	[26]
*Chrysosporium-related fungi*	horses	keratomycosis in horses	corneal ulcer, stromal abscess, or severe diffuse nonulcerative keratitis	antifungal agents locally	[25]
*C. zonatum*	horsehair	colonizing horsehair, association with *M. gypseum*	skin lesion	none/lost to follow up	[27]
*C. zonatum*	Egyptian Arabian Horses	not published	not published	not published	[50]

**Table 2:** Isolation of pathogens from horse biomaterial in Kazakhstan.

Region of Kazakhstan	Number of Samples			Micromycetes Identified			
	Total	Positive	No Growth	Total	Dermatophytes	Mold	Yeast
North Kazakhstan region	21	12	9	11	1	10	0
Akmola region	33	32	1	43	8	31	4
Kostanay region	23	7	16	20	1	19	0
Karaganda region	13	11	2	16	3	10	3
Total	90	62	28	90	13	70	7

## Data Availability

The original contributions presented in the study are included in the article; further inquiries can be directed to the corresponding author.
